# Pregametogenesis: The Earliest Stages of Gonad and Germline Differentiation in Anuran Amphibians

**DOI:** 10.3390/biology13121017

**Published:** 2024-12-05

**Authors:** Maria Ogielska, Magdalena Chmielewska, Beata Rozenblut-Kościsty

**Affiliations:** Amphibian Biology Group, Department of Evolutionary Biology and Conservation of Vertebrates, Faculty of Biological Sciences, University of Wrocław, Sienkiewicza 21, 50-335 Wrocław, Poland; maria.ogielska@uwr.edu.pl (M.O.); magdalena.chmielewska@uwr.edu.pl (M.C.)

**Keywords:** pregametogenesis, gonocytes, gonad development, anuran amphibians, spermatogenesis, oogenesis, hybridogenesis, chromatin elimination, micronuclei, western Palearctic water frogs *Pelophylax*

## Abstract

The gonads of amphibians, like other vertebrates, consist of somatic tissues, which create a specific environment essential for the differentiation of germline cells. The earliest stages of gametogenesis still remain underexplored in anuran amphibians. We propose to introduce the term “pregametogenesis” for a specific period of gonocyte proliferation and differentiation that occurs exclusively during the early stages of gonadal development. This review shows the key steps of early gonad differentiation in anuran amphibians and further compares chromatin reorganization in gonocytes of mammals and hybridogenetic water frogs. In mammals, this phase involves resetting genomic imprinting, which is crucial for determining gene expression in offspring. In hybridogenetic *Pelophylax* water frogs, we highlight the unique phenomenon of genome elimination, where one parental subgenome is eliminated while the other is replicated. This process, occurring at the same developmental phase as imprinting in mammals, underscores the evolutionary importance of pregametogenesis. The study of amphibian gonocytes provides valuable insights into chromatin reorganization and genome plasticity, offering new perspectives on reproductive biology.

## Simple Summary

Gametogenesis is the crucial process of forming egg cells and sperm in the ovaries and testes, ensuring the continuation of species and sustaining populations. In this review, we introduce the term “pregametogenesis” to describe the differentiation of pre-meiotic germline cells, known as gonocytes, which are exclusively present in the early stages of gonad development. We compare key processes in mammalian and frog gonocytes, highlighting the differences that shape the fate of these cells. In mammals, the focus is on resetting the genomic imprinting inherited from parents, which regulates gene activity in the offspring. In hybrid water frogs (*Pelophylax*), we explore the unique process of genome elimination, which occurs during the same pregametogenesis phase as imprinting in mammals. This suggests that chromatin reorganization during pregametogenesis is a critical evolutionary event in germ cell formation across vertebrates. Basic research on gonocytes could reveal insights into genome plasticity and chromatin dynamics. Gonocytes are unique cells, and their extraordinary potential may offer new avenues for advancements in regenerative medicine.

## 1. Introduction

The amphibian gonads, like in other vertebrate and invertebrate animals, are composed of somatic tissues and germline cells. Germline cells are essential for reproduction, but they cannot function independently and must cross-talk with various somatic cells that build different compartments of the gonad and provide a specific environment (niche) for differentiating germ cells (for review, see [1,2,3]). The somatic tissues of the gonad are descendants of coelomic epithelium and mesenchymal cells of mesonephric origin. The germline cells in frogs originate from specific cells situated in the endoderm that will form the primitive gut (archenteron) far from the somatic gonad primordia, to which they must immigrate (for review, see [4,5,6,7]). These cells are called primordial germ cells (PGCs). After settling into the forming gonads, PGCs divide mitotically to produce numerous daughter cells, which we call gonocytes (G). This earliest period of gametogenesis involving PGCs and gonocytes occurs only in tadpoles and juveniles before the onset of meiosis and does not occur in adult organisms, which have oocytes in the ovaries or SSCs, spermatocytes, and spermatids in the testes. For this reason, we propose the name pregametogenesis for the earliest stages of gonads development [8,9].

Gametogenesis starts after sexual differentiation of the gonads and is different in females (oogenesis) and males (spermatogenesis). Proper gametogenesis, characteristic of adult males and females, is preceded by a unique period for which we propose the name pregametogenesis, which takes place only in tadpole and juvenile gonads before the onset of meiosis. This developmental phase of gametogenesis in amphibians remains underexplored, and thereby, we adapted the better-studied mammalian models for comparison with the findings obtained in anuran amphibians so far. In males, this phase, known as prespermatogenesis, is significantly more distinctive than the corresponding preoogenesis phase in females. Although the fundamental process of gametogenesis is consistent across different animals, the nomenclature for germ cells at various developmental stages is not standardized. The following names are in use for gonocytes: primary spermatogonia and primary oogonia, gonial cells (male and female), or germinal stem cells (GSCs); these names are often confused or used interchangeably (for review, see [7,10]). The most universal and oldest name introduced originally for mammals [11] is gonocyte, and this name will be used throughout this review. This variation arises from differing practices in various laboratories and the diversity of species studied. In order to accurately describe the changes in germline cells over time and space, we begin by presenting the successive stages of gonad formation and differentiation.

## 2. Development of Gonads

### 2.1. Undifferentiated Gonad

**Figure 1:**
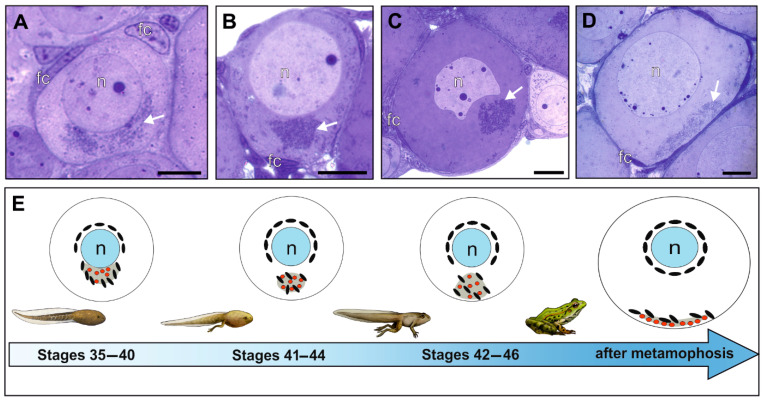
Formation of the Balbiani body in diplotene oocytes of water frogs, (**A**,**D**) *P. ridibundus*, (**B**,**C**) *P. lessonae*. (**A**) In the youngest oocytes, a mitochondrial cloud and nuage material (white arrow) form near the oocyte nucleus. (**B**) The mitochondrial cloud then assumes a spherical shape. (**C**) By the end of the metamorphosis, the migration of the Balbiani body towards the vegetal pole of the oocyte is clearly visible. (**D**) In juvenile individuals, mitochondria and germ granules are located at the vegetal pole of the oocyte as germplasm. (**E**) Schematic representation of the formation and migration of the Balbiani body, as shown in (**A**–**D**) in relation to somatic developmental stages, according to Gosner [18]. Black ovals represent mitochondria, some of which remain around the cell nucleus and do not become part of the Balbiani body. Red dots denote germ granules, and nuage is indicated in gray. (**A**–**D**) Semithin sections stained with methylene blue; white arrow—Balbiani body; fc—follicular cell; n—nucleus. Scale bar 10 µm.

**Figure 2:**
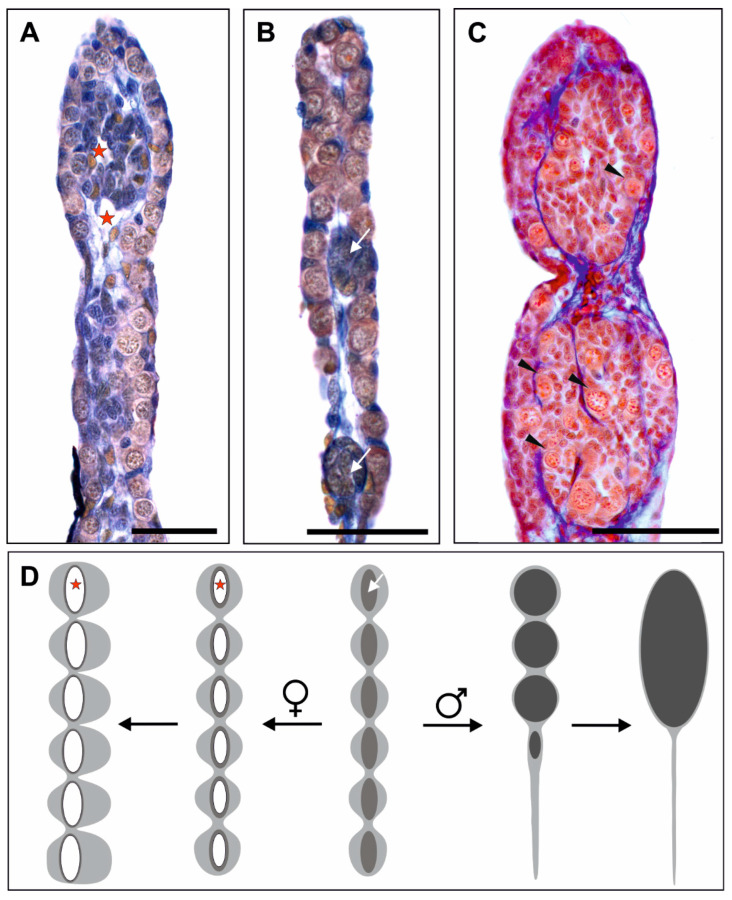
Morphology of gonads during sexual differentiation. (**A**) In the differentiating ovary, an ovarian cavity (red asterisk) appears in the somatic medulla, while gonocytes remain in the cortical region, *P. ridibundus* Gosner stage 26. (**B**) In the undifferentiated gonad, germ cells are located in the cortical region, and metamerically arranged clusters of somatic cells (knots) appear in the center, reflecting the number of gonomeres, *P. perezi* Gosner stage 25. (**C**) In the differentiating testis, gonocytes (black arrowheads) migrate to the medulla of the gonad, *P. esculentus* Gosner stage 35. (**D**) Diagram illustrating the main features of the ovary and testis during sexual differentiation, as shown in (**A**–**C**). The white arrow indicates medullary knots in gonomeres, as shown in (**B**). In testes, after differentiation, the medulla (black) transforms into seminiferous tubules, and distal gonomers degenerate. Scale bar 100 µm.

The first morphological sign of gonad formation is the appearance of genital ridges, namely two parallel longitudinal folds of monolayer coelomic peritoneum epithelium in the dorsal part of the body cavity (coeloma) along both sides of the gut mesentery. These epithelial somatic cells are reinforced by desmosome-like junctions and tight junctions that persist throughout the whole period of gonadal development in both sexes [12]. The two sheets of the epithelium form the mesenthery that suspends gonads to the body cavity; it will develop into mesovarium in females or mesorchium in males. Soon after the gonadal ridges are formed, the primordial germ cells (PGCs) migrate into their lumen. PGCs in frogs and toads are big cells filled with densely packed yolk platelets, the spherical or ovoid nucleus with a smooth nuclear envelope. PGCs have arisen from precisely defined (determined) blastomeres of the vegetative hemisphere of the early embryo that contain the specialized material known as the germplasm. Only cells containing germplasm have the information to initiate and control meiosis. The germplasm consists of mitochondria and electron-dense material in the form of granules, granulo-fibrillar material, and intermitochondrial cement. After the second cleavage (four blastomeres), the three consecutive divisions are asymmetric and segregate the germplasm into only one of the daughter cells. Germplasm is characteristic of the germline cells from PGCs to the oocytes at the pachytene stage. In the male germline cells, it is shed during spermiogenesis together with the cytoplasm. In the female germline, it is formed de novo during diplotene and is transmitted to the eggs and the embryos that arise from them; in this sense, germplasm is transgenerational and immortal. In diplotene oocytes, germplasm is composed of proliferating mitochondria arranged in a group (cloud) around centrioles and granulo-fibrillar electron-dense material (nuage) (Figure 1A,B). Nuage and mitochondria together form the more or less distinct transient structure known as the Balbiani body (Figure 1C). At late previtellogenesis, the Balbiani body moves to the vegetal pole of the oocyte and disintegrates into small islands (Figure 1D). In this context, the Balbiani body is continuous with the germplasm (Figure 2), although their composition of mRNAs and other macromolecules differs during ontogenesis. These structures have been most extensively studied in *Xenopus laevis* (Daudin, 1802) (for review, see [13,14,15,16,17]). The electron-dense material emerges from the oocyte nucleus and forms the nuage, closely apposing the nuclear membrane. It consists of RNAs, ribonucleoproteins (mRNP), and RNA-binding proteins (Figure 1E); the RNAs include those involved in germline development, such as *vasa*, *dazl*, and *nanos1* (formerly *Xcat2*), as well as non-coding RNAs, while among the protein components are members of the piRNA, which protects the integrity of the germline genome by attacking RNA generated by transposons. RNAs involved in germplasm formation (*nanos1*, *Xdazl*, *Xwnt11*, *Xlsirts*, *Hermes*, *Germes*, *Fatvg*, *Grip2*, *Xpat*, *Xvelo1*, *Xpat,* and others) are arranged in a precise spatiotemporal pattern. *Xdazl* is involved in the promotion of meiosis; it interacts with various mRNAs to control their translation, ensuring that the proteins necessary for meiosis are produced at the right time [13,14,15,16,17]). It should not be forgotten that the gonocytes of the male germline also contain the Balbiani bodies (inherited from PGCs) that initiate and control meiosis. However, the Balbiani bodies are not created de novo, as in oocytes, but are inherited from PGCs and are lost during spermiogenesis together with the cytoplasm of spermatids.

At gastrulation, presumptive PGCs move passively together with the endodermal mass to the archenteron and then actively leave the forming gut, as was evidenced both in vitro and in vivo [19,20]. They form filipodia or bleb-like protrusions, and their movement is guided by fibronectin in the gut mesenthery and regulated by the modulation of E-cadherin expression levels in the epithelium, allowing PGCs to move if the level of E-cadherin is reduced [12,21,22,23] In the mesentery, PGCs move along the defined path over the inner surface (basal lamina) of the epithelial cells and then are oriented towards the site of gonadal ridges formation. PGCs are the cells that differentiate last in the developing frog embryo, as indicated by the presence of yolk platelets, which clearly distinguish them from the surrounding tissues. As a result of the mitotic divisions during migration, approximately 60 PGCs are formed, and approximately 30 of them inhabit each genital ridge [24]. The genital ridges, together with gonocytes, form the gonadal anlage (for review, see [7,25]).

After settling in the genital ridge cortex, the PGCs stop forming pseudopodia, lose yolk by vitellolysis and transform into gonocytes [7,10,11]. In the gonadal anlage, epithelial cells start proliferating, cross the interrupted basal lamina, and give rise to the main somatic parts of a gonad, such as the outer cortex and the inner medulla, both underlined by their own basal laminae. The medulla in most of the anuran species forms groups of cells interconnected by desmosome-like junctions and arranged metamerically along the gonad (Figure 2B) [8]. These groups were originally described by Witschi [26], who named them “knots”. More recently, Fujitani et al. [27] described the medullar metameres in *Xenopus laevis* as a “mass-in-line” structure. At this time, the external metameric structure of the gonad becomes visible. The number of metameres (in gonads named gonomeres) is species-specific and ranges from less than ten in *Bufotes viridis* (Laurenti, 1768)*, Rana temporaria* (Linnaeus, 1758), *Pelophylax lessonae* (Camerano, 1882), *P. ridibundus* (Pallas, 1771), and *Bombina bombina* (Linnaeus, 1761), to more than twenty in *Xenopus laevis* [8,28]. The most anterior gonomere differentiates into the fat body [8,28]. A unique situation is observed in toads of the family Bufonidae, where the second gonomere differentiates into an ovarian lobe in both sexes, known as Bidder’s organ (for review, see [7]). Due to a localized increase in retinoic acid (RA) concentration, the germline cells within Bidder’s organ acquire the ability to enter meiosis earlier than in the rest of the gonad [29].

The cortex and the medulla are separated by space, which soon becomes invaded by mesenchymal cells (stroma) emerging from the mesonephroi [8,30]. The fibroblast-like stromal cells are negative for E-cadherin and will give rise to connective tissue that will constitute an internal scaffold of the gonad containing melanophores, blood vessels, muscle cells, and steroidogenic cells (Leydig in males or thecal in females). Stroma remains permanently separated from the medulla and the cortex epithelia by their basal laminae. Gonocytes and their descendants are also separated from the stroma by the cells derived from the coelomic epithelium, i.e., pre-Sertoli cells in the testes and prefollicular cells in the ovaries [12].

### 2.2. Sexual Differentiation of Gonads

In the female gonads, the gonocytes (also called primary oogonia) stay in their initial attachment to the gonadal surface epithelium in the cortex while the medulla remains sterile (Figure 2A). On the contrary, gonocytes in the male gonads leave the cortex and penetrate the medullar knots (Figure 2C). From this time on, gonads differentiate into the ovaries or testes. Piprek et al. [12] observed sex-specific changes in the distribution of the cell junctions during the sexual differentiation of Xenopus gonads. In the ovarian cortex, numerous desmosome-like junctions are present between somatic cells, while in the developing testes, the cell junctions disappear. This implies that in the differentiating ovaries, the adhesion of germ cells to the gonadal surface epithelium may ensure the germ cells’ persistence in their original cortical position.

### 2.3. The Ovaries

The gonomeres of the undifferentiated gonad develop into ovarian lobes (also named ovarian sacs), so the number of lobes is the same as the number of gonomeres [8] (Figure 2D). In the earliest ovaries, the stroma is well-differentiated, contains loosely dispersed collagen fibers, and is separated from the cortex and medulla by their basal laminae. The cells building the medullary knots lose their cell junctions and delaminate, which results in the formation of the intramedullary lumen lined by a monolayer of the medulla-derivative cells [12] (Figure 2A). The lumen is traditionally called the secondary ovarian cavity as opposed to the primary gonadal cavity of the undifferentiated gonad before the invasion of somatic epithelial cells, which will form the medullar knots [31].

**Figure 3:**
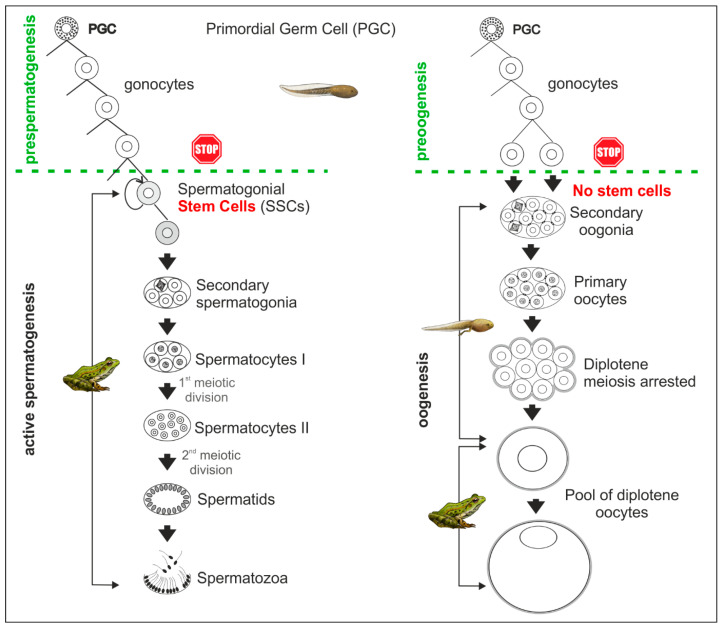
Comparison of the sequence of events during spermatogenesis (**left** side) and oogenesis (**right** side) in anurans. The green line indicates the completion of prespermatogenesis and preoogenesis. It is worth noting that stem cells are present only in adult males, whereas in oogenesis, most germ cells undergo differentiation into oocytes during the tadpole and juvenile. For more details, see the text.

The period of the most intensive proliferation of gonocytes starts immediately after the sexual differentiation of an ovary. Dividing gonocytes are located in the peripheral part of the ovarian cortex. The cortical layer of primary oogonia becomes thinner and then loses continuity and transforms into patches (called germ patches) that persist in the ovaries of adult females [8]. The mitotic divisions of gonocytes are asynchronous and decrease as the female matures. After the last mitotic division of oogonial cells, they enter a specific mitotic division without cytokinesis. The resulting cells (now called secondary oogonia) are connected by cytoplasmic bridges and are separated from the stroma by flat somatic cells that are derivatives of the prefollicular cells. In this way, secondary oogonia become encapsulated by the precursors of follicular cells joined by the desmosome-like junctions, thus forming cysts around groups (nests) of secondary oogonia. After the last mitotic cycle, the secondary oogonia remain in the cyst, transform into primary oocytes, and enter meiosis. The cytoplasmic bridges disappear at late pachytene, and meiosis is halted at the diplotene stage of meiotic prophase I. Oocytes stay at the diplotene stage of meiotic prophase I until ovulation (metaphase I) and fertilization (metaphase II) (Figure 3). The prefollicular cells of a cyst wall proliferate and encompass each diplotene oocyte by a monolayer of follicular cells; from this time on, diplotene oocytes become individual cells, and the cyst ceases its existence, giving rise to ovarian follicles. The growing number of diplotene cells protrudes into the ovarian cavity, which results in the addition of two more layers around the fully developed diplotene oocytes: a sheet of cavity epithelium underlined by a thin stromal layer [12,32]. Finally, the ripe oocytes are surrounded by three layers of somatic cells: follicular, stromal, and external epithelium.

The presence of germ patches in adult females has been considered evidence of the constant renewal of the oocyte pool in frogs [31,33]. However, our quantitative studies on the grass frog *Rana temporaria* show that a definite number of diplotene oocytes is established during the tadpole and juvenile periods of the female [34]. The final number of oocytes in a female forms a stock for 11–12 breeding seasons and exceeds the number of eggs produced during the potential reproductive life span of this species. Although the primary oogonia located in the germ patches in adult female frogs can proliferate, they fail to differentiate further, and thereby, there is no supplementation of new generations of oocytes after each spawning. More generally, the resident oogonia should not be regarded as stem cells in the oogenesis of mature female anuran amphibians (Figure 3).

### 2.4. The Testes

The first morphological sign of sexual differentiation into a testis is the displacement of germ cells and epithelial pre-Sertoli cells from the cortical region into the medulla [12,25,35,36] (Figure 2C). Unlike the ovary, not all gonomeres of the undifferentiated gonad develop into the testis (Figure 2D). Only the 3–4 proximal gonomeres differentiate into the functional male gonad, while the distal part degenerates by apoptosis, leading to a considerable shortening of the testis [35]. The external epithelium of the cortex, now devoid of germ cells, and the medulla fuse and differentiate into the external capsule, i.e., the *tunica albuginea*. The epithelial-derived cells form the seminiferous cords, and pre-Sertoli cells encapsulate the gonocytes and separate them from the stroma by the basal laminae. The cords surrounded by mesenchymal cells elongate and widen and finally give rise to the seminiferous tubules [37].

In tadpole testes, we found gonocytes in stages corresponding to those described for mammals, and therefore, we adapted the nomenclature to amphibians [38]. At the beginning of prespermatogenesis, the germline cells form a population in transition from PGCs to gonocytes with high mitotic activity (in mammals named T1- or M-prospermatogonia, respectively). After this period, gonocytes stop proliferating and enter a mitotical quiescence period as Q-prospermatogonia and stay arrested at the G0/G1 phase of the cell cycle. When the quiescent period ends, the Q-prospermatogonia enters a second wave of proliferation as T2-prospermatogonia. After the second mitotic wave stops, T2-prospermatogonia stay quiescent until puberty, when they differentiate into spermatogonial stem cells (SSCs) (for details, see [39,40,41]). However, it should be noted that the M, Q, and T2 periods are not clearly separated and overlap in time, which makes research difficult. In the western Palearctic water frogs *Pelophylax ridibundus* and *P. lessonae* from Poland [42,43], gonocytes cease proliferating and enter a quiescent phase (equal to Q-prospermatogonia) around the time of metamorphosis completion. Upon reaching sexual maturity at two years of age, these gonocytes (equal to T2-prospermatogonia) transform into spermatogonial stem cells (SSCs), which will continuously renew throughout the male’s adult life [38] (Figure 3). Before spermatogenesis reaches full activity, the precocious “first wave” of spermatogenesis occurs in both frogs [37,38,44,45] and mammals [46,47].

The seminiferous tubules in adult testes are filled with cysts in which active spermatogenesis occurs (Figure 3). In non-amniotes, such as fish and amphibians, Sertoli cells retain the ability to undergo mitotic divisions, which allows for an increase in the number of these cells in the wall of the seminiferous tubule and growing cysts during active spermatogenesis in sexually mature males [48,49]. A cyst wall is formed by Sertoli cells which surround each of the SSCs, enveloping dividing secondary spermatogonia and later spermatocytes [38,50]. After the cyst disintegrates and the spermatozoa are released, the Sertoli cells that formed the cyst apparently undergo degeneration (referred to in [51,52,53]). In contrast, in amniotes, including mammals, a constant pool of postmitotic Sertoli cells exists in the seminiferous tubules of adult males [6,48]. After several mitotic cycles of SSCs in frogs, the progenitor cells (progenitor spermatogonia according to Wang et al. [54]) become committed to the next round of specific mitoses without cytokineses giving rise to secondary spermatogonia connected by cytoplasmic bridges, corresponding to A and B spermatogonia in mammals. After the last mitotic cycle, secondary spermatogonia transform into primary spermatocytes and enter meiosis. The cytoplasmic bridges disintegrate only in the spermatid stage. The number of secondary spermatogonia cell cycles varies and ranges from 3 to 8 in *Pelophylax lessonae* and *P. ridibundus* [38], from 6 to 8 in *Bombina bombina,* and from 5 to 7 in *B. variegata* (Linnaeus, 1758) [50]. The number of Sertoli cells that constitute a single cyst is not permanent and is strongly correlated with the number of germ cells, which, in turn, is correlated with the male’s age. The mean number of germ cells per one Sertoli cell was the same, irrespective of the cyst volume, and was 6.5 ± 2.2 in *B. bombina* and 7.40 ± 2.96 in *B. variegata* [50].

### 2.5. Gonadal Development in Relation to Somatic Development

**Figure 4:**
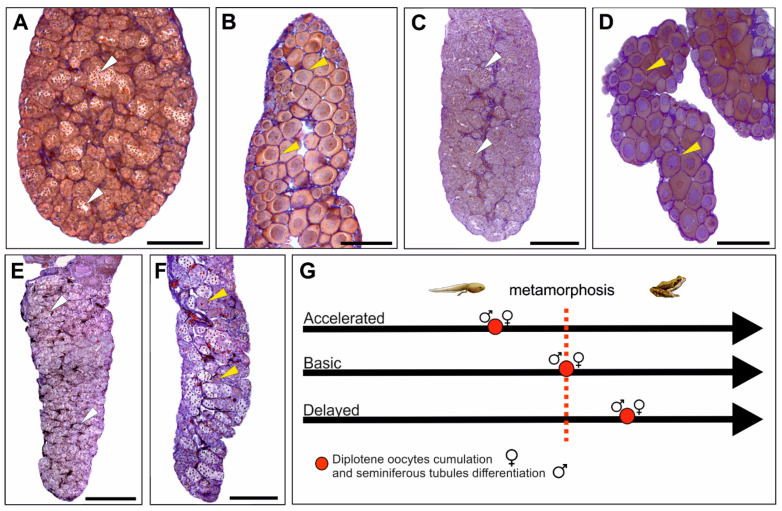
The rate of gonadal differentiation in amphibians in relation to somatic development. Accelerated in the testes (**A**) and ovaries (**B**) of *Pelophylax perezi*, basic in the testes (**C**) and ovaries (**D**) of *Hyla arborea*, and delayed in the testes (**E**) and ovaries (**F**) of *Bufotes viridis*. All gonads come from individuals just after completing metamorphosis. White arrowheads indicate the stage of development of seminiferous tubules, and yellow arrowheads indicate diplotene oocytes in the ovaries; scale bar 100 µm. (**G**) A schematic representation of the timing of oocyte pool formation in the ovaries and the differentiation of seminiferous tubules in testes in relation to the time of metamorphosis completion.

In this review, we presented the sequence of gonadal developmental events in anuran amphibians without referring to the somatic development of an individual because differences in the timing between soma and germline are species-specific. While the rate of gonad development is similar across species, the somatic developmental rate and the time of metamorphosis vary greatly. These differences mean that the metamorphosed young individuals of various species have gonads that are at different stages of development (the “gonadal clock” is constant, while the “somatic clock” is variable; for details, see [8]). This variation is the result of selective pressure from the environment in which the tadpoles develop. If the completion of metamorphosis (Gosner stage 46) [18] is the reference point of somatic development, three rates of gonad differentiation are observed for both ovaries [8] and testes [9]: basic, delayed, and accelerated (Figure 4). Ovaries developing at a basic rate show the first appearance of diplotene oocytes (Figure 4D); in ovaries developing at a delayed rate (Figure 4F), secondary oogonia appears, while in ovaries developing at an accelerated rate (Figure 4B), the cortex is composed of a growing number of diplotene oocytes. In the testes, the rates are determined by the timing of seminiferous tubule differentiation: at completion of metamorphosis (basic) (Figure 4C), in juveniles after completion of metamorphosis (delayed) (Figure 4E), or at the metamorphic climax (accelerated) (Figure 4A).

### 2.6. Genetic and Hormonal Control of Gonad Sexual Differentiation

The amphibian sex is determined genetically with female (in the ZZ/ZW system) or male (in the XX/XY system) heterogametic sex chromosomes. More than 90% of frog species examined so far have morphologically indistinguishable (homomorphic) sex chromosomes. The homomorphic sex chromosomes probably allow the replacement of a sex-determining gene with a new gene, which is unlikely in differentiated (heteromorphic) sex chromosomes in amniotes that are restricted to a particular sex-determining gene [55].

Little is known about amphibian sex-determining genes except for *dm-w* and *dmrt1,* which are master genes of gonadal sex differentiation in *X. laevis* [27,56,57,58,59]. The gene *dm-w* (DM domain-containing W-link) encodes a transcription factor characterized by the presence of a DNA-binding domain called the DM domain. It is located on the W chromosome and acts as a female sex-determining (anti-testis) gene [57]. Furthermore, *dm-w* inhibits *Dmrt1* (doublesex and mab-3 related transcription factor 1) and thereby prevents testicular differentiation. *Dmrt1* is an evolutionarily conserved gene that plays a master role in male sex determination in vertebrates, leading to testes development. The *Dmrt1*-driven masculinizing system has been conserved during vertebrate evolution [60]. In *X. laevis*, *Dmrt1* is transcribed in the nuclei of both female and male gonocytes with diffused chromatin; later in development, it appears in the secondary oogonia and spermatogonia and in the Sertoli cells.

The following genes belonging to various pathways were examined as probably being involved in the control of gonad development in *X. laevis*: *Gata4*, *Sox9*, *Dmrt1*, *Amh*, *Fgf9*, *Ptgds*, *Pdgf*, *Fshr*, and *Cyp17a1* showed an upregulation of expression in the testes, while *DM-W*, *Fst*, *Foxl2*, and *Cyp19a1* were upregulated in the ovary [59]. Shortly thereafter, Piprek et al. [61] published an in-depth analysis of *Xenopus* transcriptomes with hundreds of transcripts that emerge during gonad development. The analysis of the gene expression levels in ZW and ZZ gonads showed significant differences between the sexes and revealed a sexually dimorphic pattern of gene expression, as well as a profound difference in the gene expression between *Xenopus* and other vertebrates.

Gonad differentiation is highly dependent on sex hormones. *Cyp17a1* encodes cytochrome P450 17A1 that converts progestagens to androgens, and the steroidogenic activity is higher in developing testes than in the ovaries. The *Cyp19a1* encodes the aromatase responsible for testosterone conversion to estradiol and is expressed both in the testes and ovaries but is strongly upregulated in the ovaries. This indicates that estradiol is probably synthesized in the developing ovaries from the onset of their differentiation [62]. In *Xenopus laevis*, gonadal sex differentiation is reached at stage 56, according to Nieuwkopp and Faber [63], equivalent to stages 38–40, according to Gosner [18]. There is an asynchronous production of estradiol in genotypic female (before stage 50) and male (after stage 50) tadpoles. In females, estradiol inhibits the differentiation of the medullary cells, PGCs remain in the cortex and an ovary is formed. In genotypic males, estradiol is not produced before stage 50, and male PGCs and medullary cells differentiate into a testis [64]. The environment created by somatic cells determines the fate of germ cells. Sex steroids primarily influence the somatic cells of the gonads while having only a minor role in affecting germ cells. The prefollicular cells in the cortex enable meiosis, while pre-Sertoli cells of the medulla inhibit it [65].

Most amphibians are gonochoristic, meaning that their gonads differentiate directly into the ovaries or testes without transitional forms. Numerous experiments have shown that epigenetic factors can override the genetic sex determination in amphibians. The sexual differentiation of gonads can be altered by external factors, such as exogenous steroid hormones or xenohormones (environmental hormone disruptors) [66]. Xenohormones are by-products of agriculture, the chemical industry, and pharmaceutics and are present as contaminants in aquatic environments where amphibian eggs and tadpoles develop. The effect of xenohormones on gonadal sex differentiation depends on the time when they are absorbed by a tadpole’s body and on the rate of gonad differentiation (basic, delayed, or accelerated) in a species (Figure 1). In *X. laevis,* sex reversal may be induced before the sexual differentiation of gonads; if estrogen is applied during sex differentiation, it results in hermaphrodite gonads, and if later, it has no effect [62,64]. Compounds with estrogenic properties (17β-estradiol, BPA, and alizarin) in *Xenopus laevis*, *Bombina bombina*, *Hyla arborea* (Linnaeus, 1758), *Bufotes viridis*, and *Rana temporaria* can lead to malformations of the gonads, particularly in males, resulting in sterile or hermaphroditic gonads and segmented testes, as well as sex reversal, where the gonadal sex does not match the genetic sex [65,66,67,68,69]. On the other hand, androgenic compounds (testosterone, 17β-trenbolone) increase mortality in *Pelophylax nigromaculatus* (Hallowell, 1861) [70], *Xenopus tropicalis* (Gray, 1864) [71], *Bufotes viridis*, and *Hyla arborea* [72]. They also cause sex reversal from female to male in *Glandirana rugosa* (*Rana rugosa*) (Temminck & Schlegel, 1838), *Rana japonica* (Boulenger, 1879), *Clinotarsus curtipes* (*Rana curtipes*) (Jerdon, 1854), and *Pelophylax nigromaculatus* [73,74,75,76]. The susceptibility to hormonal sex reversal does not depend on female or male heterogamety (ZZ/ZW or XX/XY) [65].

## 3. Gonocytes—The Key Cells of Pregametogenesis

Studies on pregametogenesis are prerequisites to understanding how genetic information is established in future gametes. Gonocytes are transitional cells that are present only in larval/fetal and juvenile/pre-pubertal gonads in amphibians and mammals, respectively. Because the completion of metamorphosis in amphibians may be considered equal to birth in mammals [77,78], we compared the period of metamorphosis (premetamorphosis, prometamorphosis, and climax) in frogs to fetal life in mammals and the completion of metamorphosis to birth. Where appropriate, we will refer to the relevant stages of gametogenesis in the much more comprehensively studied mammals, especially rodents and humans. The most intriguing phase is the Q-phase of gonocytes, both in mammals and in amphibians. Gonocytes are then arrested in the G0/G1 phase of the cell cycle, and the chromatin of their nuclei undergoes extraordinary reorganization. In mammals, genomic imprinting occurs, while in hybridogenetic hybrids, one of the parental subgenomes is eliminated.

### 3.1. Genomic Imprinting in Mammals

Genomic imprinting is the result of epigenetic regulation of the allele activity without changing the DNA sequence. There are conserved groups of specific genes that are expressed from only one allele (maternal or paternal), while the other allele is repressed (imprinted). The genes are located in clusters of 3–12 genes, and each cluster contains an imprinting control region (ICR). The canonical molecular mechanism underlying imprinting is the repressive methylation of DNA, namely the addition of the methyl (CH3) group to a cytosine. This DNA methylation in gene promotors inhibits gene expression by repressing the binding of various regulatory transcription factors. DNA methylation leads to chromatin remodeling and the formation of inactive chromatin, or heterochromatin, through the recruitment of histone deacetylases and other histone-modifying proteins. An alternative non-canonical epigenetic mechanism concerns changes in the histone 3 lysine 27 trimethylation (H3K27me3). Yet another mechanism concerns long terminal repeats (LTRs) of endogenous retroviruses (transposons) that may act as regulatory elements. The imprinted genes are not numerous and display species-specific regulation patterns across mammals. There are about 200 imprinted genes in humans and mice, which constitute less than 1% of all genes. Although few in number, they are key regulators not only of fetal and postnatal development but also of adult behavior (for review, see: [79,80,81]). Somatic cells retain the methylation pattern inherited at fertilization and established in the early embryo. Throughout an individual’s life, genomic imprints are preserved and protected from the epigenetic changes occurring in somatic cells. In contrast, the imprint is erased in germline cells during pregametogenesis and is re-established in the later stages of germ cell development.

Each gamete carries its own sex-specific imprinting pattern. After the fusion of the gametes, a zygote arises with a new pattern of imprinted genes. This indicates that imprints are created anew in each individual of the new generation. The imprinting pattern is sex-specific and differs in oocytes and sperm. Genomic imprints established during pregametogenesis remain unchanged within one generation, from the time they are set in an individual’s germ cells until they are erased in the gamete precursors of their offspring. Therefore, genomic imprinting is an example of transgenerational epigenetic inheritance.

It is best to trace genomic imprinting in the gonocytes of the male germline cells during prespermatogenesis, where the process is distinctive and well-documented. The erasure of the methylation pattern inherited at fertilization, i.e., the DNA demethylation, is believed to be the most widespread and complete among cells of a developing embryo. It starts when the PGCs migrate towards and reach the genital ridges and is completed in M-prospermatogonia. This is followed by a wave of global re-methylation responsible for paternal imprinting and transposon silencing, which occurs and is completed in the Q-prospermatogonia. The T2-prospermatogonia with the new methylation pattern transforms into SSCs, which give rise to spermatocytes producing spermatozoa after meiosis and spermiogenesis. The timing of DNA methylation reprogramming is conserved across mammals, both eutherians and marsupials, over long evolutionary timescales (for review, see: [39,40,81,82]). Genomic imprinting occurs also in oogenesis. Obata and Kono [83] demonstrated that in mouse oogenesis, maternal imprinting occurs during the phase of dividing mitotic primary oogonia that corresponds to M-prospermatogonia in males. The de novo methylation that is restricted to Q-prospermatogonia in males in females extends over the diplotene oocyte growth. In mice, female germ cells begin to enter meiosis, while male M-prospermatogonia continue to undergo mitosis until they stop dividing and enter mitotic arrest as Q-prospermatogonia [84].

### 3.2. Genome Elimination in Hybridogenetic Frogs

Another example of extraordinary chromatin reorganization in gonocytes comes from hybridogenetic hybrids amphibians, the water frogs of the genus *Pelophylax* [42,43,85]. It is generally accepted that hybrid animals are infertile because the two different chromosome sets (subgenomes), one from the mother and one from the father, are not fully compatible due to inter-species diversity. In germline cells, two different chromosome sets do not interact properly during meiosis, leading to abnormal and non-functional gametes and sterility. However, it should be noted that both parental genomes function in harmony in somatic cells of viable hybrids. Studies on the structure of gonads and the course of meiosis in various hybrids were conducted mainly in the second half of the last century. In amphibians, the research involved the newt genera *Triturus* and *Lissotriton* by Callan and Spurway [86], Benazzi [87], Mancino [88], and Mancino et al. [89,90,91], and in the frogs of the genus *Lithobates* [92,93]. All these hybrids displayed gonad dysgenesis, defective germline cells with chromosomal rearrangements, and germ cell loss.

**Figure 5:**
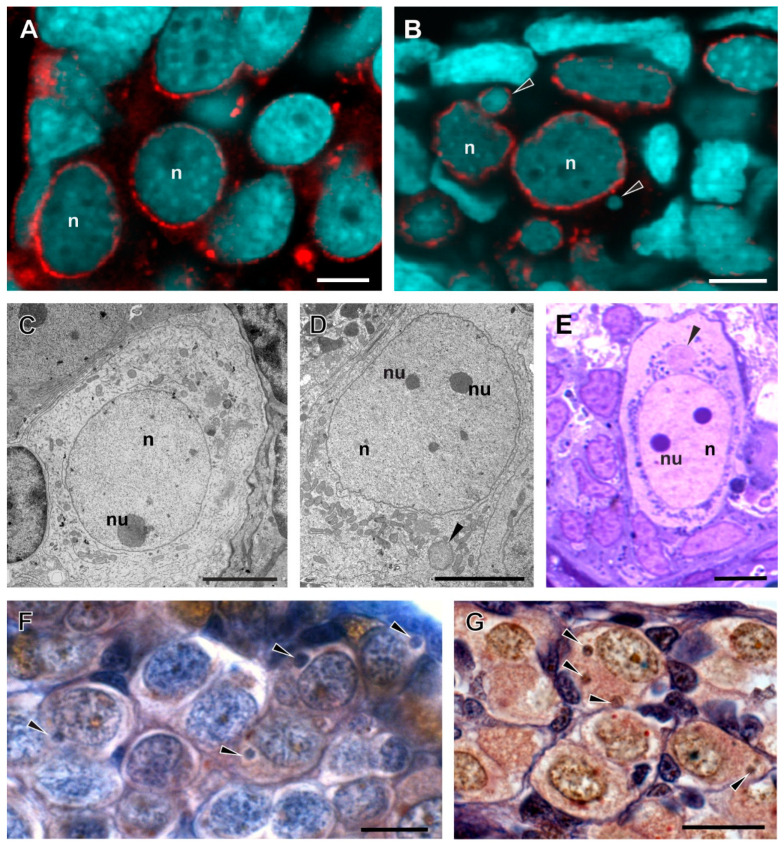
Morphology of Q-gonocytes in prespermatogenesis (**A**,**C**–**E**) and preoogenesis (**B**,**F**) in various taxa of water frogs. (**A**,**C**) Highly dispersed chromatin in nuclei of gonocytes in *P. ridibundus*, and in hybrids *P. esculentus* (**D**,**E**,**G**) and *P. grafi* (**B**,**F**); in hybrids, chromatin elimination occurs in the form of micronuclei (black arrowhead). (**A**) Male *P. ridibundus*, Gosner stage 30. (**B**) Female *P. grafi*, Gosner stage 38. (**A**,**B**) Immunofluorescent staining of frozen tissue sections, DNA stained with DAPI (blue), nuclear pore complexes (red). (**C**) Male *P. ridibundus* after metamorphosis, TEM. (**D**) Male *P. esculentus* after metamorphosis, TEM. Part of this photo was used in Chmielewska et al. 2018, as Figure 1B, published under a CC BY 4.0 license (http://creativecommons.org/licenses/by/4.0/ (accessed on 22 November 2024)). (**E**) Juvenile male *P. esculentus*, TEM. (**F**) Female *P. grafi*, Gosner stage 27. (**G**) Male *P. esculentus*, Gosner stage 40. (**F**,**G**) Mallory staining. n—nucleus, nu—nucleolus, black arrowhead—micronucleus. Scale bars: (**A**–**E**) 5 µm and (**F**,**G**) 20 µm.

Interestingly, some rare hybrids are able to remove one of the parental subgenomes from the germline cells, enabling the remaining subgenome to undergo meiosis. This unusual mode of reproduction is called hybridogenesis (for review, see [94,95,96,97]) and was originally described in the female hybrid fish *Poeciliopsis lucida-monacha* by Schultz [98]. Among amphibians, it occurs in both sexes of the European water frogs, the edible frog *Pelophylax esculentus* (Linnaeus, 1758), which is a hybrid between *P. lessonae* and *P. ridibundus*, and the Graf’s frog, *P. grafi* (Crochet, Dubois, Ohler & Tunner, 1995), the hybrid between *P. ridibundus* and *P. perezi* (López-Seoane, 1885) [99,100]. Recently, we studied genomic compositions and genome elimination processes in *P. esculentus* from Poland and *P. grafi* from the southeast of France, which both represent the western Palearctic *Pelophylax* [42,43,101]. Diploid somatic cells of hybrids possess two sets of homologous chromosomes: one from the mother (usually *ridibundus*) and one from the father (*lessonae* or *perezi*) [102,103]. Gonocytes of these hybrids eliminate one of the parental chromosomal sets (usually paternal) and reduplicate the remaining one to restore the diploid state. Both of these processes occur exclusively in interphase gonocytes during pregametogenesis in both male and female gonocytes. One of the subgenomes is eliminated inside the nuclear buds enclosed by a double membrane that detaches from the nucleus as micronuclei [85,104] (Figure 5B,D–G). These micronuclei exhibit a depletion of nuclear pore complexes in their membranes and undergo chromatin silencing and heterochromatinization. The formation of micronuclei does not trigger apoptotic cell death, indicating that genome elimination is a physiological process. Micronuclei within the cytoplasm are enclosed by separation membranes; their nuclear envelopes rupture and they are ultimately degraded via autophagy. Chromatin reorganization in gonocytes, resulting in the elimination of one subgenome and the reduplication of the other, is pivotal in understanding the mechanisms of hybridogenesis.

Genome elimination is not precise in all gonocytes. Sometimes, not all chromosomes are removed, leading to aneuploidy of gonocytes and later gametes [105,106]. Even when elimination occurs properly, subgenome reduplication fails to take place in some gonocytes, providing compelling evidence that these two processes are independent. This results in the formation of haploid gonocytes that cannot undergo normal meiosis. We estimated that elimination and reduplication occurred correctly in only about 20% of gonocytes, which subsequently transformed into SSCs in mature *P. esculentus* males [36].

### 3.3. Genomic Imprinting in Mammals and Genome Elimination in Hybrid Frogs Both Occur During the Same Phase of Pregametogenesis

Gonocytes have a long arrest time of the cell cycle in many animals. In the mouse, Q-prospermatogonia remain halted in the G0/G1 phase until 3–4 days after birth, when they transform into T2-prospermatogonia and reinitiate high mitotic activity [84]. The pause duration in the hamster is 8 days; in the rat—10 days; in the teleost *Oryzias latipes* (Temminck and Schlegel, 1846)—15 days; in the rabbit—7 weeks; in the ram and cow—2 months (reviewed in [107]); and in the *Pelophylax* frogs—a minimum of 46 days [38]. Genomic imprinting has not been studied in amphibian gonocytes. However, our findings on the elimination of one chromosome set in the interphase gonocytes of hybridogenetic hybrids suggest that a type of chromatin reorganization may also occur in amphibians. What caught our attention was the highly dispersed chromatin in the nuclei of interphase gonocytes in frogs (Figure 5A,C,D) [38,104], which is strikingly similar to that described in mammalian Q-spermatogonia in the G0/G1 phase [41,107,108].

Yoshioka et al. [84] followed the patterns of DAPI and constitutive heterochromatin-specific markers, the histone H3 trimethylation of lysine 9 (H3K9me3), and the non-histone protein CBX5 in the mouse prespermatogenesis. They reported the loss of heterochromatin aggregations (chromocentres) in Q-prospermatogonia, suggesting a direct link between entry into mitotic arrest at the G0/G1 stage and the appearance of diffuse chromatin. The authors also suggested that the dynamic changes in heterochromatin reorganization contribute to epigenetic reprogramming of the paternal genome in fetal prospermatogonia and represent a unique epigenomic state that is particularly amenable to reprogramming.

Some naturally occurring hybrids provide unexpected opportunities to observe the earliest stages of gametogenesis during sexual differentiation of the gonads. In hybridogenetic frogs, such as *P. esculentus* [85,104,109] and *P. grafi* (*status: manuscripts in preparation* [110,111], as well as in hybridogenetic fish *Hypseleotris* [112,113], micronuclei appear in interphase gonocytes with highly dispersed chromatin, suggesting that they are in the G0/G1 phase and are equivalent to Q-prospermatogonia in mammals. Our observations of interphase gonocytes in hybridogenetic frogs lead us to accept the thesis that genome elimination occurs in the G0/G1 phase. *Pelophylax esculentus* and *P. grafi* are very good models for studying the Q-gonocytes because they are relatively easy to observe both during spermatogenesis and oogenesis. Moreover, the period of gonocyte proliferation is extended in hybrids as compared to the parental species due to imprecise genome elimination. As a result, the gonocyte population, particularly the Q-gonocytes, remains accessible to researchers for a significantly longer duration [36,114].

**Figure 6:**
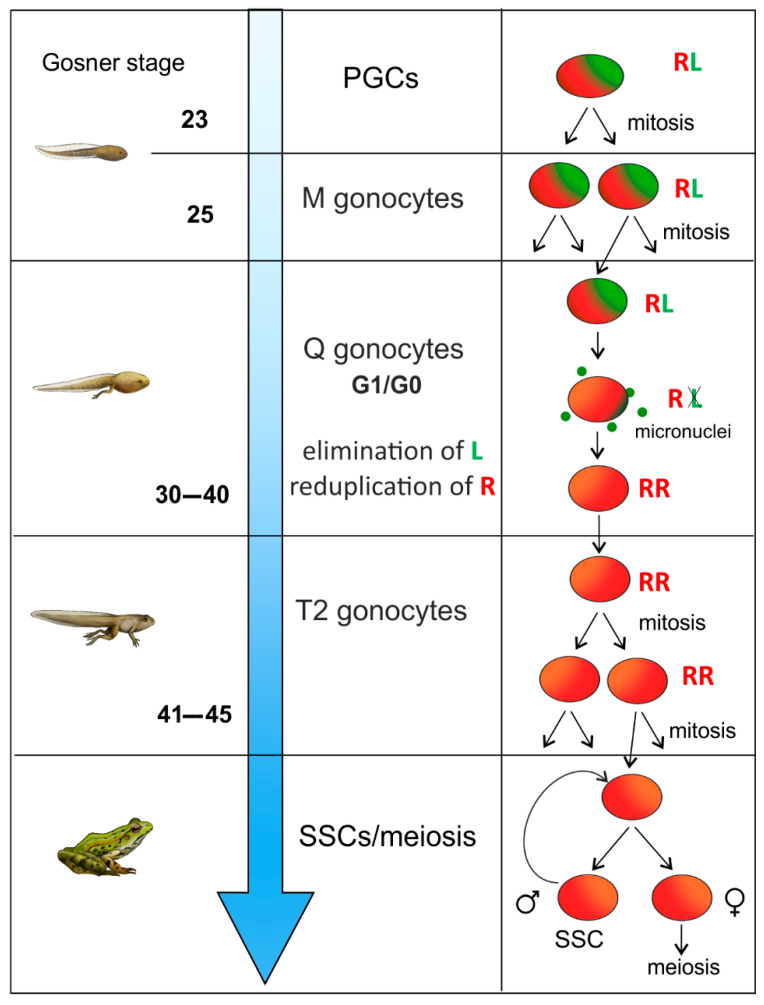
The process of elimination and endoreplication during pregametogenesis in hybridogenetic edible frogs *Pelophylax esculentus*. Initially, PGCs and M-gonocytes have a hybrid chromosomal composition (R + L genomes). At the Q-gonocyte stage, one subgenome (L) is eliminated from the cell nuclei as micronuclei (green dots), which are degraded, while the remaining R subgenome undergoes endoreplication to the diploid RR state. T2-gonocytes then undergo mitotic divisions, increasing their number. This is followed by gonocyte differentiation into SSC-type stem cells in adult males, while meiosis begins in females. Somatic development corresponds to the Gosner developmental scale.

Direct evidence for the chromatin reorganization in male and female gonocytes in the Q phase was provided by studies of their chromosomal composition. In male *P. esculentus*, PGCs and gonocytes corresponding to M-prospermatogonia have *ridibundus*-*lessonae* (R + L) chromosomes (Figure 6), the gonocytes corresponding to Q-prospermatogonia display a full spectrum of elimination and reduplication *in statu nascendi* (RL→R→RR), whereas in the gonocytes corresponding to T2-prospermatogonia, the diploid *ridibundus* (R + R) chromosomal complements were observed [36]. The results in female *P. grafi* (R + P) are similar [110].

The molecular mechanism of genome elimination is unknown. We may only speculate that the hypothetic genes (*) or transposons (*) that extort the rejection of one of the subgenomes are imprinted and silenced in somatic cells. During the time corresponding to chromatin reorganization in the Q-phase of pregametogenesis, the imprinting could be erased, allowing one of the genomes (*lessonae* or *perezi*) to be eliminated and new imprinting to occur in the remaining subgenome. Therefore, it is hypothesized that the resulting hybrid gametes transmit the imprinted *ridibundus* genome (R*) to the hybrid progeny R*L or R*P.

These two examples of extraordinary chromatin reorganization, namely genomic imprinting in mammals and genome elimination in hybridogenetic frogs, may suggest that genome remodeling in gonocytes may also occur in other taxa. Exceptions to the rule are often excellent opportunities to learn about the functioning of common processes, such as gametogenesis. However, this requires a new approach and thorough comparative research on the earliest stages of gametogenesis (pregametogenesis) in non-mammalian vertebrates.

## 4. Summary and Conclusions

The gonads of amphibians, like other vertebrates, consist of somatic tissues and germline cells, which interact to create a specific environment essential for gonadal development. Initially, the undifferentiated gonad, under the influence of genetic and hormonal factors, develops into either an ovary or a testis. As the structure of the gonad changes, germ cells differentiate into gonocytes, while somatic cells differentiate into follicular cells in females and Sertoli cells in males. The earliest phase of gametogenesis, which we propose to call “pregametogenesis”, deserves special attention. During this stage, numerous mitotic divisions increase the number of gonocytes, then cell divisions cease, and chromatin disperses in the quiescence phase (G0/G1). In this crucial period, gonocytes undergo chromatin reorganization. In mammals, this phase is characterized by the erasure of methylation patterns and the establishment of a new genomic imprinting pattern specific to gonocytes, which determines gene activity in offspring. Similarly, in hybridogenetic water frogs, we observe chromatin reorganization through the elimination of one genome and endoreplication of the other, enabling meiosis and ensuring fertility in hybrids. These two examples of chromatin modifications highlight profound genomic and cytological changes in germ cells beyond just their ability to undergo reductive division. The pregametogenesis period is underexplored in various animal groups, but we believe that fundamental studies of gonocytes across taxa may provide universal insights into developmental biology. Currently, a lively debate exists between two groups of scientists. Woods and Tilly [115] argue that active oogonial stem cells are absent in the mature mouse ovary, while Yoshihara and colleagues [116] contend that these cells are indeed present. The progress of regenerative medicine opened the discussion because oogonial stem cells, if present, are essential in the treatment of infertility [117]. Today, regenerative medicine is exploring gonocytes in the context of extended or impaired fertility in humans, making these studies highly relevant for future medical applications.

## References

[1] Spradling A., Drummond-Barbosa D., Kai T. (2001). Stem cells find their niche. Nature.

[2] Lehmann R. (2012). Germline Stem Cells: Origin and Destiny. Cell Stem Cell.

[3] Papagiannouli F., Lohmann I. (2015). Stage-specific control of stem cell niche architecture in the Drosophila testis by the posterior Hox gene Abd-B. Comput. Struct. Biotechnol. J..

[4] Ogielska M., Bartmańska J., Ogielska M. (2009). Oogenesis and Female Reproductive System in Amphibia—Anura. Reproduction of Amphibians.

[5] Ogielska M., Bartmańska J., Ogielska M. (2009). Spermatogenesis and Male Reproductive System in Amphibia-Anura. Reproduction of Amphibians.

[6] Roco Á.S., Ruiz-García A., Bullejos M. (2021). Testis Development and Differentiation in Amphibians. Genes.

[7] Ogielska M., Ogielska M. (2009). The Undifferentiated Amphibian Gonad. Reproduction of Amphibians.

[8] Ogielska M., Kotusz A. (2004). Pattern and rate of ovary differentiation with reference to somatic development in anuran amphibians. J. Morphol..

[9] Goldberg J. (2015). Gonadal Differentiation and Development in the Snouted Treefrog, *Scinax fuscovarius* (Amphibia, Anura, Hylidae). J. Herpetol..

[10] Culty M. (2013). Gonocytes, from the Fifties to the Present: Is There a Reason to Change the Name?. Biol. Reprod..

[11] Clermont Y., Perey B. (1957). Quantitative study of the cell population of the seminiferous tubules in immature rats. Am. J. Anat..

[12] Piprek R., Kloc M., Tassan J.-P., Kubiak J. (2017). Development of Xenopus laevis bipotential gonads into testis or ovary is driven by sex-specific cell-cell interactions, proliferation rate, cell migration and deposition of extracellular matrix. Dev. Biol..

[13] Berekelya L.A., Mikryukov A.A., Luchinskaya N.N., Ponomarev M.B., Woodland H.R., Belyavsky A.V. (2007). The protein encoded by the germ plasm RNA Germes associates with dynein light chains and functions in Xenopus germline development. Differentiation.

[14] Juliano C., Wang J., Lin H. (2011). Uniting Germline and Stem Cells: The Function of Piwi Proteins and the piRNA Pathway in Diverse Organisms. Annu. Rev. Genet..

[15] Nijjar S., Woodland H.R. (2013). Localisation of RNAs into the Germ Plasm of Vitellogenic *Xenopus* Oocytes. PLoS ONE.

[16] Jamieson-Lucy A., Mullins M.C. (2019). The vertebrate Balbiani body, germ plasm, and oocyte polarity. Curr. Top. Dev. Biol..

[17] Kloc M., Bilinski S., Chan A.P., Allen L.H., Zearfoss N.R., Etkin L.D. (2001). RNA localization and germ cell determination in *Xenopus*. Int. Rev. Cytol..

[18] Gosner K.L. (1960). A Simplified Table for Staging Anuran Embryos Larvae with Notes on Identification. Herpetologica.

[19] Wylie C.C., Roos T.B. (1976). The formation of the gonadal ridge in *Xenopus laevis*. III. The behaviour of isolated primordial germ cells in vitro. J. Embryol. Exp. Morphol..

[20] Wylie C.C. (1980). Primordial Germ Cells in Anuran Embryos: Their Movement and Its Guidance. Bioscience.

[21] Heasman J., Hynes R.O., Swan A.P., Thomas V., Wylie C.C. (1981). Primordial germ cells of Xenopus embryos: The role of fibronectin in their adhesion during migration. Cell.

[22] Dzementsei A., Schneider D., Janshoff A., Pieler T. (2013). Migratory and adhesive properties of Xenopus laevis primordial germ cells in vitro. Biol. Open.

[23] Baronsky T., Dzementsei A., Oelkers M., Melchert J., Pieler T., Janshoff A. (2016). Reduction in E-cadherin expression fosters migration of Xenopus laevis primordial germ cells. Integr. Biol..

[24] Hardisty M.W. (1967). The numbers of vertebrate primordial germ cells. Biol. Rev..

[25] Piprek R.P., Kubiak J.Z., Małgorzata K., Kubiak Jacek Z. (2014). Development of Gonads, Sex Determination, and Sex Reversal in *Xenopus*. Xenopus Development.

[26] Witschi E. (1929). Studies on sex differentiation and sex determination in amphibians. I. Development and sexual differentiation of the gonads of *Rana sylvatica*. J. Exp. Zool..

[27] Fujitani K., Otomo A., Wada M., Takamatsu N., Ito M. (2016). Sexually dimorphic expression of Dmrt1 and γH2 AX in germ stem cells during gonadal development in *Xenopus laevis*. FEBS Open Bio.

[28] Piprek R.P., Pecio A., Kloc M., Kubiak J.Z., Szymura J.M. (2014). Evolutionary trend for metamery reduction and gonad shortening in Anurans revealed by comparison of gonad development. Int. J. Dev. Biol..

[29] Piprek R.P., Pecio A., Laskowska-Kaszub K., Kloc M., Kubiak J., Szymura J. (2013). Retinoic acid homeostasis regulates meiotic entry in developing anuran gonads and in Bidder’s organ through Raldh2 and Cyp26b1 proteins. Mech. Dev..

[30] Falconi R., Dalpiaz D., Zaccanti F. (2004). Ultrastructural aspects of gonadal morphogenesis in *Bufo bufo* (Amphibia Anura) 1. Sex differentiation. J. Exp. Zool. A Comp. Exp. Biol..

[31] Witschi E. (1929). Studies on sex differentiation and sex determination in amphibians. III. rudimentary hermaphroditism and Y chromosome in Rana temporaria. J. Exp. Zool..

[32] Piprek R.P., Kloc M., Kubiak J.Z., Piprek R.P. (2016). Early Development of the Gonads: Origin and Differentiation of the Somatic Cells of the Genital Ridges. Results and Problems in Cell Differentiation.

[33] Witschi E. (1956). Amphibian ovogenesis. Development of Vertebrates.

[34] Ogielska M., Kotusz A., Augustyńska R., Ihnatowicz J., Paśko Ł. (2013). A Stockpile of Ova in the Grass Frog *Rana temporaria* is Established Once for the Life Span. Do Ovaries in Amphibians and in Mammals Follow the Same Evolutionary Strategy?. Anat. Rec..

[35] Haczkiewicz K., Ogielska M. (2013). Gonadal sex differentiation in frogs: How testes become shorter than ovaries. Zool. Sci..

[36] Chmielewska M., Kaźmierczak M., Rozenblut-Kościsty B., Kolenda K., Dudzik A., Dedukh D., Ogielska M. (2022). Genome elimination from the germline cells in diploid and triploid male water frogs *Pelophylax esculentus*. Front. Cell Dev. Biol..

[37] Bartmańska J., Ogielska M. (1999). Development of testes and differentiation of germ cells in water frogs of the Rana esculenta-complex (Amphibia, Anura). Amphib. Reptil..

[38] Haczkiewicz K., Rozenblut-Kościsty B., Ogielska M. (2017). Prespermatogenesis and early spermatogenesis in frogs. Zoology.

[39] McCarrey J.R. (2013). Toward a More Precise and Informative Nomenclature Describing Fetal and Neonatal Male Germ Cells in Rodents. Biol. Reprod..

[40] Ishihara T., Griffith O.W., Tarulli G.A., Renfree M.B. (2021). Male germline development in the tammar wallaby, *Macropus eugenii*. Reproduction.

[41] Wrobel K.H. (2000). Prespermatogenesis and spermatogoniogenesis in the bovine testis. Anat. Embryol..

[42] Plötner J. (2005). Die Westpaläarktischen Wasserfrösche: Von Märtyrern der Wissenschaft zur Biologischen Sensation.

[43] Dufresnes C., Monod-Broca B., Bellati A., Canestrelli D., Ambu J., Wielstra B., Dubey S., Crochet P., Denoël M., Jablonski D. (2024). Piecing the barcoding puzzle of Palearctic water frogs (*Pelophylax*) sheds light on amphibian biogeography and global invasions. Glob. Change Biol..

[44] Iwasawa H., Nakazawa T., Kobayashi T. (1987). Histological observations on the reproductive organs of growing *Rana nigromaculata* frogs. Sci. Rep. Niigata Univ. Ser. D.

[45] de Rooij D.G., Russell L.D. (2000). All you wanted to know about spermatogonia but were afraid to ask. J. Androl..

[46] Yoshida S., Sukeno M., Nakagawa T., Ohbo K., Nagamatsu G., Suda T., Nabeshima Y. (2006). The first round of mouse spermatogenesis is a distinctive program that lacks the self-renewing spermatogonia stage. Development.

[47] Hermann B.P., Sukhwani M., Hansel M.C., Orwig K.E. (2010). Spermatogonial stem cells in higher primates: Are there differences from those in rodents?. Reproduction.

[48] França L.R., Nóbrega R.H., Morais R.D., Assis L.H.D.C., Schulz R.W. (2015). Sertoli cell structure and function in anamniote vertebrates. Sertoli Cell Biology.

[49] Lara N.d.L.e.M., Costa G.M.J., Figueiredo A.F.A., França L.R.d. (2019). The Sertoli cell: What can we learn from different vertebrate models?. Anim. Reprod..

[50] Rozenblut-Kościsty B., Piprek R., Pecio A., Bartmańska J., Szymura J.M., Ogielska M. (2017). The structure of spermatogenic cysts and number of Sertoli cells in the testes of *Bombina bombina* and *Bombina variegata* (Bombinatoridae, Anura, Amphibia). Zoomorphology.

[51] Pudney J. (1995). Spermatogenesis in nonmammalian vertebrates. Microsc. Res. Tech..

[52] Bouma J., Cloud J.G., Skinner M.K., Griswold M.D. (2005). Sertoli Cell Biology in Fishes and Amphibians. Sertoli Cell Biology.

[53] Exbrayat J.-M., Méndez-Vilas A. (2017). From the Sertolian syncytium to Sertoli cells in anamniotes, especially Gymnophionan (Caecilian) Amphibians. Microscopy and Imaging Science: Practical Approaches to Applied Research and Education.

[54] Wang J.-H., Li Y., Deng S.-L., Liu Y.-X., Lian Z.-X., Yu K. (2019). Recent Research Advances in Mitosis during Mammalian Gametogenesis. Cells.

[55] Perrin N. (2021). Sex-chromosome evolution in frogs: What role for sex-antagonistic genes?. Philos. Trans. R. Soc. B Biol. Sci..

[56] Yoshimoto S., Okada E., Umemoto H., Tamura K., Uno Y., Nishida-Umehara C., Matsuda Y., Takamatsu N., Shiba T., Ito M. (2008). A W-linked DM-domain gene, DM-W, participates in primary ovary development in *Xenopus laevis*. Proc. Natl. Acad. Sci. USA.

[57] Yoshimoto S., Ito M. (2011). A ZZ/ZW-type sex determination in *Xenopus laevis*. FEBS J..

[58] Ito M., Mawaribuchi S. (2013). Molecular Evolution of Genes Involved in Vertebrate Sex Determination. Encyclopedia of Life Sciences.

[59] Piprek R.P., Damulewicz M., Kloc M., Kubiak J.Z. (2018). Transcriptome analysis identifies genes involved in sex determination and development of *Xenopus laevis* gonads. Differentiation.

[60] Yoshimoto S., Ikeda N., Izutsu Y., Shiba T., Takamatsu N., Ito M. (2010). Opposite roles of DMRT1 and its W-linked paralogue, DM-W, in sexual dimorphism of *Xenopus laevis*: Implications of a ZZ/ZW-type sex-determining system. Development.

[61] Piprek R.P., Damulewicz M., Tassan J.-P., Kloc M., Kubiak J.Z. (2019). Transcriptome profiling reveals male- and female-specific gene expression pattern and novel gene candidates for the control of sex determination and gonad development in *Xenopus laevis*. Dev. Genes Evol..

[62] Ito M., Kobayashi K., Kitano T., Iwao Y., Kondo M. (2018). Sex Determination and Differentiation in Frogs. Reproductive and Developmental Strategies, Diversity and Commonality in Animals.

[63] Nieuwkoop P.D., Faber J. (1994). Normal Table of Xenopus laevis (Daudin): A Systematical and Chronological Survey of the Development from the Fertilized Egg Till the End of Metamorphosis.

[64] Villalpando I., Merchant-Larios H. (1990). Determination of the sensitive stages for gonadal sex-reversal in *Xenopus laevis* tadpoles. Int. J. Dev. Biol..

[65] Piprek R.P., Pecio A., Kubiak J.Z., Szymura J.M. (2012). Differential effects of testosterone and 17β-estradiol on gonadal development in five anuran species. Reproduction.

[66] Hayes T.B. (1998). Sex determination and primary sex differentiation in amphibians: Genetic and developmental mechanisms. J. Exp. Zool..

[67] Tamschick S., Rozenblut-Kościsty B., Ogielska M., Kekenj D., Gajewski F., Krüger A., Kloas W., Stöck M. (2016). The plasticizer bisphenol A affects somatic and sexual development, but differently in pipid, hylid and bufonid anurans. Environ. Pollut..

[68] Tamschick S., Rozenblut-Kościsty B., Ogielska M., Lehmann A., Lymberakis P., Hoffmann F., Lutz I., Kloas W., Stöck M. (2016). Sex reversal assessments reveal different vulnerability to endocrine disruption between deeply diverged anuran lineages. Sci. Rep..

[69] Tamschick S., Rozenblut-Kościsty B., Ogielska M., Lehmann A., Lymberakis P., Hoffmann F., Lutz I., Schneider R.J., Kloas W., Stöck M. (2016). Impaired gonadal and somatic development corroborate vulnerability differences to the synthetic estrogen ethinylestradiol among deeply diverged anuran lineages. Aquat. Toxicol..

[70] Li Y.-Y., Xu W., Chen X.-R., Lou Q.-Q., Wei W.-J., Qin Z.-F. (2015). Low concentrations of 17β-trenbolone induce female-to-male reversal and mortality in the frog *Pelophylax nigromaculatus*. Aquat. Toxicol..

[71] Olmstead A.W., Kosian P.A., Johnson R., Blackshear P.E., Haselman J., Blanksma C., Korte J.J., Holcombe G.W., Burgess E., Lindberg-Livingston A. (2012). Trenbolone causes mortality and altered sexual differentiation in *Xenopus tropicalis* during larval development. Environ. Toxicol. Chem..

[72] Rozenblut-Kościsty B., Ogielska M., Hahn J., Kleemann D., Kossakowski R., Tamschick S., Schöning V., Krüger A., Lutz I., Lymberakis P. (2019). Impacts of the synthetic androgen Trenbolone on gonad differentiation and development–comparisons between three deeply diverged anuran families. Sci. Rep..

[73] Shirane T. (1986). A new, early, morphological indication of sex differentiation in anura, *Rana japonica* and *R. nigromaculata*. J. Exp. Zool..

[74] Saidapur S.K., Gramapurohit N.P., Shanbhag B.A. (2001). Effect of Sex Steroids on Gonadal Differentiation and Sex Reversal in the Frog, *Rana curtipes*. Gen. Comp. Endocrinol..

[75] Okada G., Maruo K., Funada S., Nakamura M. (2008). Differential display analysis of gene expression in female-to-male sex-reversing gonads of the frog *Rana rugosa*. Gen. Comp. Endocrinol..

[76] Phuge S.K., Gramapurohit N.P. (2015). Sex hormones alter sex ratios in the Indian skipper frog, *Euphlyctis cyanophlyctis*: Determining sensitive stages for gonadal sex reversal. Gen. Comp. Endocrinol..

[77] Tata J.R. (1993). Gene expression during metamorphosis: An ideal model for post-embryonic development. BioEssays.

[78] Tata J.R. (1996). Amphibian metamorphosis: An exquisite model for hormonal regulation of postembryonic development in vertebrates. Dev. Growth Differ..

[79] Hanna C.W., Kelsey G. (2021). Features and mechanisms of canonical and noncanonical genomic imprinting. Genes Dev..

[80] Tucci V., Isles A.R., Kelsey G., Ferguson-Smith A.C., Tucci V., Bartolomei M.S., Benvenisty N., Bourc’his D., Charalambous M., Dulac C. (2019). Genomic Imprinting and Physiological Processes in Mammals. Cell.

[81] Hubert J.-N., Demars J. (2022). Genomic Imprinting in the New Omics Era: A Model for Systems-Level Approaches. Front. Genet..

[82] Bowles J., Koopman P. (2010). Sex determination in mammalian germ cells: Extrinsic versus intrinsic factors. Reproduction.

[83] Obata Y., Kono T. (2002). Maternal Primary Imprinting Is Established at a Specific Time for Each Gene throughout Oocyte Growth. J. Biol. Chem..

[84] Yoshioka H., McCarrey J.R., Yamazaki Y. (2009). Dynamic Nuclear Organization of Constitutive Heterochromatin During Fetal Male Germ Cell Development in Mice. Biol. Reprod..

[85] Chmielewska M., Dedukh D., Haczkiewicz K., Rozenblut-Kościsty B., Kaźmierczak M., Kolenda K., Serwa E., Pietras-Lebioda A., Krasikova A., Ogielska M. (2018). The programmed DNA elimination and formation of micronuclei in germ line cells of the natural hybridogenetic water frog *Pelophylax esculentus complex*. Sci. Rep..

[86] Callan H.G., Spurway H. (1951). A study of meiosis in interracial hybrids of the newt, *Triturus cristatus*. J. Genet..

[87] Benazzi M. (1954). Sulla l’ibridazione fra *Triturus vulgaris* (L.) e *Triturus italicus* (Perarcca). Monit. Zool. Ital..

[88] Mancino G. (1959). La struttura dell’ovario nell’ibrido. Ital. J. Zool..

[89] Mancino G., Ragghianti M., Bucci-Innocenti S. (1979). Experimental Hybridization within the Genus *Triturus* (Urodela: Salamandridae). II. The Lampbrush Chromosomes of F_1_ Species Hybrids Between *Triturus cristatus carnifex* and *T. vulgaris meridionale*. Caryologia.

[90] Mancino G., Ragghianti M., Bucci-Innocenti S. (1978). Experimental hybridization within the genus *Triturus* (Urodela: Salamandridae). Spermatogenesis of F 1 species hybrids, *Triturus cristatus carnifex* ♀× *T. vulgaris meridionalis* ♂. Chromosoma.

[91] Mancino G., Ragghianti M., Bucci-Innocenti S. (1979). Experimental hybridization within the genus *Triturus* (Urodela: Salamandridae). III. Evidence for crossing-over, true chiasmata and chomosomal homologies in the spermatogenesis of F1 species hybrids, *T. cristatus carnifex* ♀ x *T. marmomtus* ♂. Chromosoma.

[92] Moore J.A. (1946). Incipient intraspecific isolating mechanisms in *Rana pipiens*. Genetics.

[93] Elinson R.P. (1977). Amphibian Hybrids: A Genetic Approach to the Analysis of Their Developmental Arrest. Differentiation.

[94] Dawley R.M., Bogart J.P. (1989). Evolution and Ecology of Unisexual Vertebrates.

[95] Lamatsch D., Stöck M., Schön I., Martens K., Dijk P. (2009). Sperm-Dependent Parthenogenesis and Hybridogenesis in Teleost Fishes. Lost sex. The Evolutionary Biology of Parthenogenesis.

[96] Ogielska M., Ogielska M. (2009). Development and Reproduction of Amphibian Species, Hybrids, and Polyploids. Reproduction of Amphibians.

[97] Lavanchy G., Schwander T. (2019). Hybridogenesis. Curr. Biol..

[98] Schultz R.J. (1969). Hybridization, Unisexuality, and Polyploidy in the Teleost Poeciliopsis (Poeciliidae) and Other Vertebrates. Am. Nat..

[99] Crochet P.-A., Dubois A., Ohler A., Tunner H. (1995). Rana (Pelophylax) ridibunda Pallas, 1771, Rana (Pelophylax) perezi Seoane, 1885 and their associated klepton (Amphibia, Anura): Morphological diagnoses and description of a new taxon. Bull. Du Muséum Natl. D’histoire Nat..

[100] Berger L. (1968). Morphology of the F1 generation of various crosses within Rana esculenta complex. Acta Zool. Cracoviensia.

[101] Dufresnes C., Mazepa G. (2009). Hybridogenesis in Water Frogs. Els.

[102] Zaleśna A., Choleva L., Ogielska M., Rábová M., Marec F., Ráb P. (2011). Evidence for Integrity of Parental Genomes in the Diploid Hybridogenetic Water Frog Pelophylax esculentus by Genomic in situ Hybridization. Cytogenet. Genome Res..

[103] Dudzik A., Dedukh D., Crochet P.-A., Rozenblut-Kościsty B., Rybka H., Doniol-Valcroze P., Choleva L., Ogielska M., Chmielewska M. (2023). Cytogenetics of the Hybridogenetic Frog *Pelophylax grafi* and Its Parental Species *Pelophylax perezi*. Genome Biol. Evol..

[104] Ogielska M. (1994). Nucleus-like bodies in gonial cells of Rana esculenta [Amphibia, Anura] tadpoles—A putative way of chromosome elimination. Zool. Pol..

[105] Dedukh D., Litvinchuk S., Rosanov J., Mazepa G., Saifitdinova A., Shabanov D., Krasikova A. (2015). Optional endoreplication and selective elimination of parental genomes during oogenesis in diploid and triploid hybrid European water frogs. PLoS ONE.

[106] Bucci S., Ragghianti M., Mancino G., Berger L., Hotz H., Uzzell T. (1990). Lampbrush and mitotic chromosomes of the hemiclonally reproducing hybridRana esculenta and its parental species. J. Exp. Zool..

[107] Nagano R., Tabata S., Nakanishi Y., Ohsako S., Kurohmaru M., Hayashi Y. (2000). Reproliferation and relocation of mouse male germ cells (gonocytes) during prespermatogenesis. Anat. Rec..

[108] Baillie A.H. (1964). The Histochemistry and Ultrastucture of the Gonocyte. J. Anat. Lond..

[109] Dedukh D., Riumin S., Chmielewska M., Rozenblut-Kościsty B., Kolenda K., Kaźmierczak M., Dudzik A., Ogielska M., Krasikova A. (2020). Micronuclei in germ cells of hybrid frogs from *Pelophylax esculentus complex* complex contain gradually eliminated chromosomes. Sci. Rep..

[110] Dudzik A., Rozenblut-Kościsty B., Dedukh D., Crochet P.-A., Choleva L., Doniol-Valcroze P., Ogielska M., Chmielewska M. Is Genome Elimination from Germline Cells More Precise in the Natural Hybrid Frog Pelophylax grafi?.

[111] Rozenblut-Kościsty B., Chmielewska M., Dudzik A., Crochet P.-A., Doniol-Valcroze P., Ogielska M. Genome Elimination Mechanism is Evolutionarily Conserved in Hybrid Frogs of the Genus *Pelophylax*: Insights from Pelophylax grafi.

[112] Majtánová Z., Dedukh D., Choleva L., Adams M., Ráb P., Unmack P.J., Ezaz T. (2021). Uniparental genome elimination in Australian carp gudgeons. Genome Biol. Evol..

[113] Dedukh D., Majtánová Z., Ráb P., Ezaz T., Unmack P.J. (2024). Gradual chromosomal lagging drive programmed genome elimination in hemiclonal fishes from the genus *Hypseleotris*. Sci. Rep..

[114] Ogielska M., Wagner E. (1993). Oogenesis and ovary development in the natural hybridogenetic water frog, *Rana esculenta* L. I Tadpole stages until metamorphosis. Zool. Jahrbücher Physiol..

[115] Woods D.C., Tilly J.L. (2023). Revisiting Claims of the Continued Absence of Functional Germline Stem Cells in Adult Ovaries. Stem Cells.

[116] Yoshihara M., Wagner M., Damdimopoulos A., Zhao C., Petropoulos S., Katayama S., Kere J., Lanner F., Damdimopoulou P. (2023). The Continued Absence of Functional Germline Stem Cells in Adult Ovaries. Stem Cells.

[117] Cheng H., Shang D., Zhou R. (2022). Germline stem cells in human. Signal Transduct. Target. Ther..

